# Giant Cell Tumor of the Olecranon With a Displaced Pathological Fracture Successfully Treated by Intralesional Curettage and Plate Fixation: A Case Report

**DOI:** 10.1155/cro/1212381

**Published:** 2026-05-26

**Authors:** Toshio Kojima, Eiji Osaka, Takao Ishii, Kazuyoshi Nakanishi

**Affiliations:** ^1^ Department of Orthopaedic Surgery, Nihon University School of Medicine, Tokyo, Japan, nihon-u.ac.jp; ^2^ Department of Orthopaedic Surgery, Kasukabe Medical Center, Saitama, Japan; ^3^ Department of Orthopaedic Surgery, Kawaguchi Municipal Medical Center, Saitama, Japan, kawaguchi-mmc.org

**Keywords:** elbow, giant cell tumor of bone, intralesional curettage, olecranon, pathological fracture, plate fixation

## Abstract

Giant cell tumor of bone (GCTB) is a locally aggressive intermediate bone tumor that typically arises at the epiphysis of long bones. GCTB occurring in the olecranon is extremely rare, and the optimal surgical strategy when complicated by pathological fractures with marked displacement involving the articular surface remains unclear. In this case, the fracture site was significantly displaced. Through the gap created by the displacement, we performed thorough curettage of the lesion, anatomical reduction, synthetic bone grafting, and plate fixation. Histopathological examination confirmed GCTB. At 9 months postoperatively, the patient was pain‐free, with a full range of motion in the elbow joint and no functional limitations. The patient remained asymptomatic at 10 years postoperatively, and imaging showed no evidence of local recurrence. This case suggests that, in olecranon GCTB complicated by pathological fracture, access through the fracture gap may allow thorough intralesional curettage and anatomical reduction and fixation in certain situations, potentially leading to favorable long‐term functional and oncological outcomes. However, pathological fracture in GCTB remains a clinical challenge and should not generally be considered advantageous.

## 1. Introduction

Giant cell tumor of bone (GCTB) is an intermediate bone tumor characterized by locally aggressive behavior that typically arises in the epiphyseal region of long bones, most frequently involving the distal femur, proximal tibia, and distal radius [1, 2]. The biological behavior and radiographic extent of GCTB are commonly assessed using the Campanacci classification, which stratifies lesions based on cortical integrity and local aggressiveness [3].

For Campanacci Grade I or II lesions, particularly those confined within the bone without extensive cortical destruction or extraosseous extension, intralesional curettage is widely accepted as the standard treatment to achieve local tumor control while preserving adjacent joint function [4, 5]. However, the presence of a pathological fracture complicates treatment selection. Previous studies have shown that pathological fracture itself does not necessarily increase the risk of local recurrence but can significantly influence surgical decision‐making by affecting bone integrity, surgical exposure, and the feasibility of joint preservation [6].

When pathological fractures involve the articular surface or demonstrate marked displacement, surgeons must carefully balance joint‐preserving curettage against more aggressive resection to ensure oncological safety [7]. This dilemma becomes particularly challenging in anatomically uncommon sites.

GCTB involving the olecranon is extremely rare, with only a few cases reported in the literature [8–11]. Cases further complicated by a displaced pathological fracture involving the articular surface are even rarer, and the optimal surgical strategy in such situations remains unclear. Here, we report a rare case of olecranon GCTB with a markedly displaced pathological fracture and describe its favorable long‐term outcome following joint‐preserving intralesional surgery. This case report is presented in accordance with the CARE guidelines.

## 2. Case Report

A 29‐year‐old woman presented with sudden right elbow pain after losing her balance and falling while holding onto a bus handrail. Although the trauma was minor, physical examination revealed moderate swelling and tenderness around the elbow. Her medical history included untreated severe diabetes mellitus and obesity.

Laboratory investigations revealed a fasting blood glucose level of 302 mg/dL (reference range, 70–99 mg/dL), hemoglobin A1c of 9.6% (4.6%–6.2%), and elevated tartrate‐resistant acid phosphatase 5b (TRACP‐5b) of 520 mU/dL (120–420 mU/dL in women). Serum calcium and phosphorus levels were within normal ranges.

**Figure 1:**
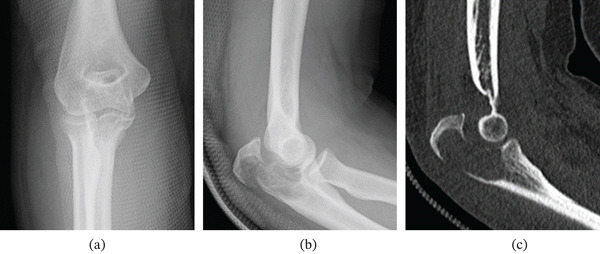
Preoperative plain radiographs and computed tomography (CT) demonstrating an osteolytic lesion of the olecranon with markedly displaced pathological fracture involving the articular surface. The size and thickness of the proximal fragment are preserved, and the normal curvature of the articular surface is disrupted. (a) Anteroposterior radiograph. (b) Lateral radiograph. (c) Sagittal CT image.

Plain radiographs and computed tomography demonstrated an osteolytic lesion with cortical destruction at the olecranon, accompanied by a pathological fracture with marked displacement and disruption of the articular surface. The size and thickness of the proximal fragment were preserved (Figure 1).

**Figure 2:**
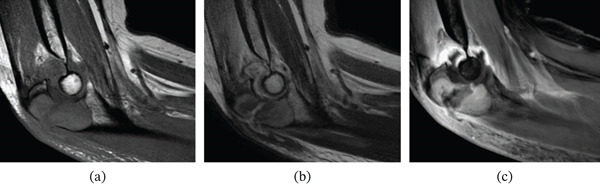
Preoperative sagittal magnetic resonance imaging of the olecranon. (a) T1‐weighted image. (b) T2‐weighted image. (c) Gadolinium‐enhanced T1‐weighted fat‐suppressed image demonstrating an intraosseous lesion without extraosseous extension.

Magnetic resonance imaging (MRI) showed a well‐demarcated solid lesion with an isointense signal on T1‐weighted images and a hyperintense signal on T2‐weighted images, with marked gadolinium enhancement. No intra‐articular extension was observed (Figure 2).

Based on imaging showing an epiphyseal lesion with cortical thinning that remained confined within bone, a Campanacci Grade I–II olecranon GCTB complicated by a pathological fracture was considered the most likely diagnosis. Elevated TRACP‐5b levels supported this diagnosis. The differential diagnoses included aneurysmal bone cyst, brown tumor, chondroblastoma, metastatic bone tumor, and osteosarcoma.

Because bone tumors of the olecranon are rare, a preoperative biopsy for histopathological diagnosis was considered. However, after comprehensively evaluating the following factors, it was determined that it was appropriate to determine the surgical strategy using intraoperative frozen section analysis without performing a bone biopsy: Percutaneous needle biopsy was technically difficult due to the small size of the lesion, there were concerns regarding the risk of infection associated with a bedside percutaneous needle biopsy due to the patient′s poorly controlled diabetes, and therapeutic surgery following an incisional biopsy would be time‐consuming, carrying a risk of malunion due to significant fracture displacement. If intraoperative frozen section diagnosis revealed no malignant findings, the plan was to perform thorough curettage and bone grafting in accordance with the treatment protocol for Campanacci Grade I or II GCTB. Furthermore, since the proximal bone fragment was of sufficient size, it was determined that fixation using an olecranon plate would be feasible. If the intraoperative frozen section diagnosis could not rule out a malignant tumor, the plan was to limit the procedure to an incisional biopsy.

**Figure 3:**
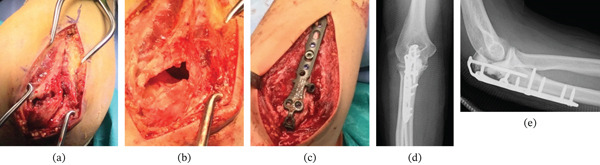
Intraoperative macroscopic findings and postoperative plain radiographs. (a) The tumor was exposed through the fracture site. Adequate visualization was obtained via the displaced fracture segment without additional cortical windowing, facilitating thorough intralesional curettage. (b) The tumor cavity after curettage. The articular cortical bone was clearly visualized through the fracture gap. Accurate anatomical reduction was achieved during temporary fixation prior to bone grafting. (c) Intraoperative photograph after definitive internal fixation using an olecranon plate, demonstrating stable fixation following curettage and reconstruction. (d) Postoperative anteroposterior plain radiograph. (e) Postoperative lateral radiograph showing satisfactory restoration of the articular surface.

Surgery was performed via a posterior approach with the patient in the lateral decubitus position. After incising the periosteum and flexing the elbow, the tumor was readily exposed through the fracture gap. Macroscopically, the lesion exposed through the fracture gap consisted of friable, brownish tissue typical of GCTB. Intraoperative frozen section diagnosis of a partial tumor specimen revealed no malignant findings. Thorough curettage was performed under adequate visualization through the fracture gap. After curettage, the articular surface was directly visualized through the cavity. Ethanol was applied as a local adjuvant therapy. The articular surface was then directly visualized, anatomically reduced, and temporarily stabilized. The resulting defect was filled with synthetic bone graft material, and definitive fixation was achieved using an olecranon plate (A.L.P.S., Zimmer Biomet) (Figure 3).

**Figure 4:**
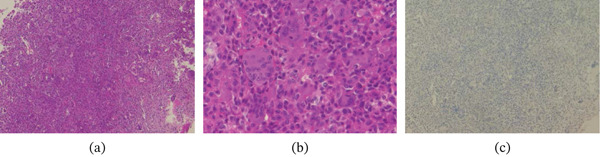
Histopathological findings of the curettage specimen. (a) Low‐power hematoxylin and eosin (H&E) staining showing multinucleated giant cells relatively uniformly distributed within a background of mononuclear stromal cells with nuclear morphology similar to that of the multinucleated giant cells (×100). (b) High‐power H&E staining demonstrating multinucleated giant cells with numerous nuclei, surrounded by mononuclear stromal cells with indistinct cytoplasmic borders and round nuclei (×400). (c) Immunohistochemical staining negative for S‐100 protein (×100).

Histopathological examination of the curettage specimen revealed uniformly distributed multinucleated giant cells within a background of mononuclear stromal cells with similar nuclear morphology, consistent with GCTB (Figure 4).

**Figure 5:**
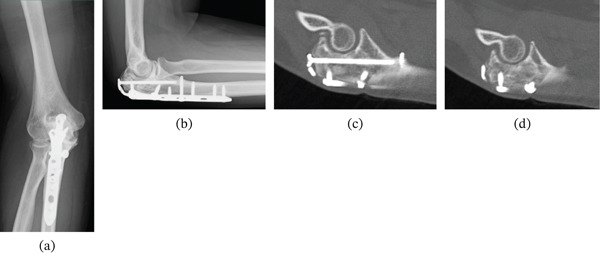
Plain radiographs and computed tomography (CT) images obtained 10 years after surgery demonstrating maintained anatomical reduction of the articular surface, stable fixation without implant failure, and no radiological evidence of local recurrence. (a) Anteroposterior radiograph. (b) Lateral radiograph. (c, d) Sagittal CT images.

Serum TRACP‐5b levels normalized within 6 months postoperatively. By 9 months after surgery, the patient was pain‐free with no limitation in elbow range of motion, no instability, and no restriction in activities of daily living. At that time, the Mayo Elbow Performance Score was 100 points, indicating an excellent elbow‐specific functional outcome, and the Musculoskeletal Tumor Society (MSTS) score was 30 points, reflecting excellent overall function following tumor surgery. At the final follow‐up, 120 months after surgery, the patient remained completely asymptomatic. Plain radiographs and computed tomography demonstrated maintained anatomical reduction of the articular surface, stable fixation, and no evidence of local recurrence (Figure 5).

## 3. Discussion

Pathological fracture represents a distinctive challenge in the management of GCTB. Although fracture itself is not an independent predictor of local recurrence, it can substantially alter surgical conditions by affecting exposure, stability, and reconstructive feasibility [7]. Consequently, fracture characteristics such as displacement and articular involvement often influence the choice between intralesional curettage and more aggressive resection [4].

There are a few reports on olecranon GCTB. To the best of our knowledge, Sanjay et al. were the first to report a detailed case of olecranon GCTB [10]. The lesion extended to the proximal end of the olecranon, and tumor resection was performed. Goyal et al. similarly reported a case in which the tumor had progressed to the proximal end of the olecranon but did not involve a pathological fracture, and resection alone was performed [8]. Minhas et al. presented a case in which reconstruction was performed using a fibular graft following en bloc excision [9]. Yang et al. reported on a GCTB of the olecranon accompanied by an incomplete fracture [11]. In this report, similar to the present case, the size of the proximal olecranon fragment was preserved, and curettage of the lesion, allografting, and plate fixation were performed. Although the patient remained recurrence‐free at 26 months postoperatively, postoperative function and imaging findings were not reported. In this case, the size of the proximal fragment, the location of the lesion, and the pathological fracture influenced the treatment strategy. The tumor cavity was widely exposed through the gap created by significant fracture displacement. Consequently, thorough curettage was performed under an adequate surgical field. Furthermore, following tumor resection and under adequate visualization, precise anatomical reduction and fixation of the articular surfaces—essential for preventing posttraumatic osteoarthritis—was performed [12]. Additionally, because the proximal fragment was large, robust fixation was achieved using an olecranon plate. Therefore, this case is noteworthy in that it suggests the possibility of performing thorough curettage and accurate anatomical reduction under adequate visualization in cases of olecranon GCTB with significant displacement and sufficient residual proximal bone fragments, as seen in this case. It should be noted that pathological fractures are generally considered adverse events in GCTB. They can complicate intralesional surgery by compromising stability and visibility. Therefore, this should not be interpreted as a beneficial outcome. The improved intraoperative visibility observed in this case was incidental and site‐specific.

Serum TRACP‐5b is a marker of osteoclast activity and is known to be elevated in various conditions, including osteoporosis, metastatic bone tumor, and GCTB [13–15]. In the present case, TRACP‐5b was elevated despite the patient′s young age, which supported the preoperative suspicion of GCTB. Although TRACP‐5b is not disease‐specific, assessment of this marker may provide useful diagnostic information in bone tumors occurring at atypical anatomical sites, where radiological features alone may be insufficient.

Intraoperative frozen section diagnosis was used to guide surgical decision‐making in this case. Previous studies have demonstrated the high diagnostic accuracy of frozen section examination in bone tumors containing lesions rich in osteoclasts, such as GCTB [16, 17]. However, particularly for bone tumors in rare locations, it is preferable to obtain a more definitive preoperative pathological diagnosis via open biopsy or needle biopsy. In this case, intraoperative rapid pathological diagnosis was used as a safety measure because the patient had severe diabetes and was at risk of infection; however, this is not a standard strategy in cases where GCTB is suspected.

Various adjuvant treatments following curettage, including high‐speed burring, chemical agents, and polymethylmethacrylate (PMMA) cementation, have been reported to reduce local recurrence in GCTB [17–19]. However, in the present case, aggressive burring was avoided because the proximal fragment was small, and excessive bone removal could have compromised temporary fixation and initial stability after plate fixation. Although PMMA cementation is known to reduce recurrence through thermal and cytotoxic effects [19, 20], its use near the articular surface carries a risk of cartilage injury due to polymerization heat [21, 22]. Despite the availability of these adjuvant techniques, previous studies have emphasized that adequate visualization of the tumor cavity and meticulous intralesional curettage are the most critical factors for minimizing local recurrence in GCTB [4, 23]. This case involved a GCTB occurring at the rare site of the olecranon. Flexing the elbow allowed for curettage with a clear view of the fracture gap. The decision to adopt adjunctive therapies should be made on a case‐by‐case basis, taking into account the stability of fixation, potential risks to articular cartilage, and site‐specific factors.

The fixation strategy was another important consideration. For displaced olecranon fractures, both tension band wiring and plate fixation are commonly employed. In the present case, cortical thinning and the postcurettage cavity raised concerns regarding loss of reduction with tension band wiring. Plate fixation was therefore selected to provide stable fixation and allow early mobilization. This approach is supported by previous studies demonstrating comparable functional outcomes between the two methods but lower rates of loss of reduction, complications, and reoperation with plate fixation [24, 25].

## 4. Conclusion

This case involved a rare GCTB of the olecranon. It was classified as Campanacci Grade I or II, with a pathological fracture present and the size of the proximal bone fragment preserved. This suggests that thorough intralesional curettage and accurate anatomical reduction and fixation may be possible under these specific circumstances. However, pathological fractures remain a clinical challenge, and caution is warranted when generalizing these findings.

## Author Contributions

T.K.: conceptualization, surgical management and procedure, and manuscript drafting; E.O.: manuscript editing and critical revision; T.I.: clinical management and surgical procedure; K.N.: supervision and final approval of the manuscript.

## Funding

No funding was received for this manuscript.

## Disclosure

All authors critically reviewed and approved the final manuscript.

## Ethics Statement

Institutional review board (IRB) approval was not required for this case report in accordance with institutional guidelines. Written informed consent was obtained from the patient.

## Consent

Written informed consent was obtained from the patient for publication of this case report and any accompanying images.

## Conflicts of Interest

The authors declare no conflicts of interest.

## Data Availability

The data that support the findings of this study are available on request from the corresponding author. The data is not publicly available due to privacy or ethical restrictions.
